# Requests for Compensation in Cases Involving Patients’ Falls in Healthcare Settings: A Retrospective Analysis

**DOI:** 10.3390/healthcare11091290

**Published:** 2023-04-30

**Authors:** Federica Mele, Mirko Leonardelli, Stefano Duma, Carlo Angeletti, Gerardo Cazzato, Carmelo Lupo, Ettore Gorini, Cristoforo Pomara, Alessandro Dell’Erba, Maricla Marrone

**Affiliations:** 1 Section of Legal Medicine, Department of Interdisciplinary Medicine, University of Bari “Aldo Moro”, 70124 Bari, Italy; 2 Section of Molecular Pathology, Department of Precision and Regenerative Medicine and Ionian Area (DiMePRe-J), School of Medicine, University of Bari “Aldo Moro”, 70100 Bari, Italy; 3 Innovation Department, Diapath S.p.A., Via Savoldini n. 71, 24057 Martinengo, Italy; 4 Department of Economics and Finance, University of Bari “Aldo Moro”, 70124 Bari, Italy; 5 Department of Medical, Surgical and Advanced Technologies “G.F. Ingrassia”, University of Catania, 95121 Catania, Italy

**Keywords:** medical malpractice, Italian legislation, patients’ falls, risk of falls, fall-related injuries

## Abstract

Falls are the most frequent adverse events recorded in healthcare facilities. By employing a multifaceted strategy to ensure prevention interventions that are specific to the patient type and environmental risk management, risk factor evaluation may help to reduce falls in the hospital setting. Patient falls are one of the main causes of lawsuits against hospitals, which has led to the development of validated instruments that are beneficial in treating the patient after the incident and effective in minimizing the frequency of falls. The aim of our study is to evaluate compensation claims asserting healthcare culpability in situations where a patient fell in a hospital setting. The collected data relate to judgments issued in Italy until December 2022 regarding 30 episodes of falls that occurred between 2003 and 2018. Our research revealed that approximately 50% of Italian healthcare organizations lose the case in court when a patient falls in a hospital setting and dies or is injured. In half of these cases, the failure of the medical staff to use protective equipment against falls is what led to the court’s acceptance of the compensation claim. In order to improve the quality of healthcare services, fall prevention techniques must continue to be implemented.

## 1. Introduction

Falls are the most common adverse event recorded among frail patients and commonly occur in elderly people that are admitted to various healthcare settings, such as acute hospitals and nursing homes [1]. It is estimated that falls affect approximately 50 percent of the elderly patients admitted to different institutional settings [2]. According to the literature, each year, an average of 1.5 falls for each patient’s bed occurs in nursing homes [3]. In the hospital environment, certain patients could be at the greatest risk of falling, for example, ischemic stroke patients, cancer patients, and patients admitted to psychiatric wards [4,5,6].

Falls occurring in the hospital environment result in a significantly increased rate of mortality and morbidity. The fall injury rate recorded varies in different studies. Some authors reported a rate of 2.36 falls per 1000 beds, while others reported a rate of 20 falls per 1000 hip fractures and 270 falls per 1000 head injuries, with 13 percent that required medical attention [7,8]. One percent of the falls among hospitalized elderly people result in a fracture, with a further 5 percent resulting in trauma requiring medical care [9,10]. Elderly people who reported a femur fracture as a result of a fall in a hospital environment are more likely to die from complications after the injury than the general population [11]. The risk factors responsible for falls in the hospital environment can be divided into intrinsic factors related to the patient’s health condition and extrinsic factors related to the environmental, ergonomic, and organizational aspects of care. Some studies reported that older age and deterioration of the mental state are intrinsic risk factors for falls [12,13]. Patients with gait disturbances, weakness, or dizzy episodes should be considered at more serious risk than others [3,14]. 

In addition, among the intrinsic risk factors related to falls, taking drugs that affect the state of alertness and balance, such as neuroleptics and benzodiazepines, as well as substances that reduce blood pressure, such as vasodilators, diuretics, and beta-blockers, should be considered [15,16]. Among the extrinsic factors, the most relevant are the following: slippery floors due to the absence of anti-slip solutions, beds or stretchers that cannot be adjusted in height, toilets without supports when lifting, and the inadequate size of the in-patient rooms [17,18].

The adoption of instruments to assess the risk of falls in hospitals could be very useful. Among these, the use of assessment tools for the prediction of the risk of patients falling in healthcare facilities could be effective in the prediction and prevention of these events among patients [19]. The most significant characteristics of these specific tests are the following: simple execution, short duration, and repeatability of the test to allow for follow-up. The scientific literature shows that these characteristics are fulfilled by the timed up and go test, the Morse fall scale (MFS), the St. Thomas’s risk assessment tool in falling elderly inpatients (STRATIFY), the Hendrich II fall risk model (HFRM), and the Tinetti balance test [20,21,22,23,24].

Risk factor assessments could help to reduce falls in the hospital environment by implementing a multifactorial strategy, in order to guarantee prevention interventions related to the type of patient and to environmental risk management [25]. A review of the literature showed that the interventions that have been shown to be most effective in reducing falls in the hospital environment are the following: mobilization and ambulation, environmental adaptation strategies, a review of drug therapy, and appropriate staff training [26].

Conversely, it has been demonstrated that physical restraint is not associated with a reduction in the risk of falls [27]. Restraint is only acceptable when it is a necessary component of therapy and should only be used when absolutely necessary [28].

Internationally, from a medico-legal point of view, patient falls during healthcare provision are one of the primary sources of litigations in hospitals, which has led to the creation of validated tools that are effective in reducing the incidence of falls (such as mobilization and ambulation, drug therapy reviews, and adequate staff training) and that are useful in managing the patient’s care after the event [29,30].

A reduction in the risk of falling in the hospital environment is an indicator of the quality of care provided [31]. 

With this aim, in Italy, the Ministry of Health endorsed Recommendation No. 13, “Prevention and management of patient falls in healthcare facilities”, addressed to all healthcare facilities and to all healthcare operators, in order to protect patients [32]. Due to the growing number of legal disputes, it is appropriate for the medical examiner to be part of the technical commission for fall prevention in the hospital environment. For these reasons, the Italian Supreme Court of Cassation (an institution at the apex of ordinary jurisdiction) established that healthcare facilities have an obligation to safeguard the physical safety of their patients (Penal Cassation, Section 4, sentence passed on 17 May 2013, No. 21285).

In cases of alleged medical fault regarding the consequences of the fall (i.e., the injury or death of the patient), the hospital litigated against must prove that the fall was attributable to unforeseeable conduct of the patient, thereby constituting a fortuitous event capable of interrupting the causal link (Italian Civil Cassation, sentence passed on 6 June 2017, No. 14037). The healthcare facility must be able to prove that it has put all the appropriate procedures in place to reduce the risk of its patients falling.

This paper focuses on the Italian context. The objective of this study is to assess the claims for compensation for alleged healthcare liability regarding events in which a patient’s fall in a hospital environment resulted in their death or injury. This has been achieved by analyzing the most frequent clinical context, the types of risk factors, what behavior, if any, can be attributed to the defending healthcare facility, and, above all, the reasons for accepting or rejecting the claim for compensation. 

An analysis of these factors could help to identify preventive strategies in order to reduce falls in hospital environments and, as a consequence, to reduce the requests for compensation and, thus, public expenditure. Furthermore, from a risk management perspective, the identified preventive strategies could contribute to improving patient safety. For this reason, our analysis could be useful for both forensic physicians and risk managers working in hospitals, as well as all healthcare professionals working in roles related to patient management in hospital settings.

## 2. Materials and Methods

For this study, we carried out a retrospective analysis searching for judgments issued in Italy until December 2022 concerning claims for compensation regarding death or injury resulting from the patient’s fall in healthcare settings. We used two different Italian databases recording Ministry of Justice sentences: “One Legale” and “De Jure Giufrè”. Since the present paper is based on an analysis of the judgments, the study necessarily conforms to a retrospective design. Furthermore, the retrospective analysis of any context could help in the improvement of future approaches regarding the same issue.

The study sample was set up randomly, selecting 44 judgments using the keywords “healthcare liability” and “hospital falls”. The individual judgments were then analyzed and those concerning falls that occurred in non-hospital settings (nursing homes and assisted-living facilities) were excluded from the study. From an initial sample of 44 sentences (100%), 14 (31.82%) were removed from the analysis after the application of those exclusion criteria. Of these sentences, 30 of the judgments analyzed (68.18%) concerned falls in the hospital environment that occurred in the time interval between 2003 and 2018. The collected data were then broken down in the following order: intrinsic risk factors related to the patient’s pathological state, the manner of the fall’s occurrence, consequences of the fall, the outcome of the dispute along with the reasons for the acceptance or rejection of the claim, and compensation for damages.

This is an observational retrospective study. Therefore, our hospital ethics committee was not involved, and informed consent was not necessary.

The data disaggregation is based on the information retrieved from the judgments and is related to the major factors cited in the requests for compensation. The statistical analysis was conducted using the Microsoft Excel 2013 software (Microsoft Corporation, Redmond, WA, USA) and the IBM Statistical Package for the Social Sciences (SPSS Statistics) version 25 for Windows (IBM Corporation, Armonk, NY, USA).

## 3. Results

The data collected in this study relate to judgments issued in Italy and concern 30 episodes of falls occurring in a hospital environment between 2003 and 2018. All the judgments were passed by the Civil Courts of First Instance in various Italian cities.

Although our research was conducted regarding judgments issued until December 2022, no sentences emerged relating to episodes of falls in a hospital environment that occurred after 2018. This is probably due to the fact that the Italian Civil Code provides for the possibility of bringing professional liability lawsuits between 5 and 10 years following the event. Among the intrinsic fall risk factors recorded, a homogeneous distribution was observed between neurological disorders (N = 10; 33.33%), advanced age (N = 8; 26.67%), cognitive impairment (N = 7; 23.33%) and motor deficits (N = 6; 20%). In 6 cases (20%), the patient had no pathological condition that increased the risk of falling. Conversely, in judgments concerning injuries secondary to the fall, a higher prevalence of femur fractures (N = 11; 36.66%) was observed, followed by polytraumas (N = 6; 20%) and head traumas (N = 6; 20%), pelvis fractures (N = 3; 10%), vertebral traumas (N = 2; 6.66%), upper limb fractures (N = 1; 3.33%), and knee traumas (N = 1; 3.33%) (Table 1).

The manner of fall recorded included falls while the patient was lying down (N = 20; 66.66%), falls while the patient was going to the toilet (N = 6; 20%), and falls while the patient was sitting on a chair/in an armchair (N = 4; 13.33%). 

**Figure 1:**
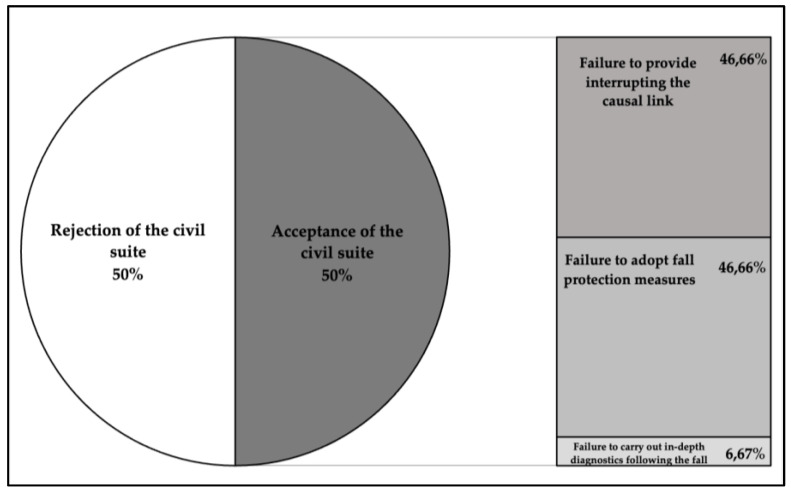
Reasons for the acceptance of civil suits.

From an evaluation of the judgments, a 50% acceptance value of the plaintiff’s claim emerged; in the remaining 50%, the claim was rejected because the fall was considered an accidental event. The reasons for the acceptance of the civil suit are summarized in Figure 1, where three different macro-groups of reasons are reported: failure to provide evidence regarding the existence of a fortuitous event interrupting the causal link (N = 7; 46.66%) (e.g., the absence of witness data regarding the dynamics of the incident), failure to adopt fall protection measures (in most cases, no rails were attached to the bed) (N = 7; 46.66%), and failure to carry out in-depth diagnostics following the fall (N = 1; 6.67%) (Figure 1).

When analyzing the group of 15 sentences in which the liability of the defendant healthcare facility was acknowledged, it was observed that the defendant was sentenced to pay an average value of EUR 27,982.55 for non-material damage (ranging from a minimum value of EUR 774.08 to a maximum value of EUR 114,695.95, depending on the extent of bodily damage). Finally, the sentences were divided into two groups pre- and post-Ministerial Recommendation No. 13, one concerning those events prior to December 2011 (9 cases) and one concerning those events after December 2011 (21 cases). For each group, the absolute value and percentage of the acceptance and rejection of each claim for compensation was calculated, as shown in Table 2.

## 4. Discussion

Elderly people are often prone to falls in the hospital environment; this represents the main factor that threatens their independence and autonomy. Falls often occur when the compensatory capacity of the elderly is impaired, as is the case with geriatric syndromes [33]. Falls in the elderly correlate with high rates of morbidity and the risk of death. For example, a large retrospective study conducted in Brazil showed that elderly patients aged 80 years and over had a more statistically significant major risk of mortality from falls than other patients [34]. In addition, several studies have found that some elderly patients would rather die than suffer hospitalization as a result of a fall [35]. 

Furthermore, in the USA, it has been estimated that the implementation of fall prevention activities could save up to USD 440 million annually in medical costs, underlining the importance of fall prevention [36]. In fact, in the USA, it is estimated that the costs associated with fatal falls are approximately USD 754 million, while the costs associated with nonfatal falls range from approximately USD 30 billion to USD 50 billion [37].

Internationally, several studies have been conducted in order to identify preventive strategies, including educational initiatives aimed at optimizing drug therapies in hospital environments, such as the more cautious use of loop diuretics, digitalis and digoxin, beta-blockers, and the so-called “Z-drugs” (comprising zolpidem, zoplicone, eszoplicone, and zaleplon) [38,39]. In addition, although an association between depression and the risk of falls is postulated, some authors also reported a relationship between selective serotonin reuptake inhibitors (SSRIs) and an increased risk of falls [40,41].

Some interventions were aimed at reducing the incidence of a single risk factor, while in others, several risk factors were addressed simultaneously. In general, the scientific literature shows that preventive actions are more effective when they are tailored to the individual patient, based on his or her own intrinsic risk factors [42]. For example, the application of a fall prevention protocol, based on a risk assessment of each patient and patient education regarding prevention strategies, seems to be effective in the reduction of falls and related injuries [43].

However, a single intervention aimed at increasing the patient’s walking autonomy has been shown to significantly reduce the incidence of falls, as reported by a meta-analysis that included 192 studies on elderly subjects who were older than 75 years [44].

Although fall prevention programs have been shown to be effective in controlled research settings, their practical application in real-world care settings has not led to the same results in terms of a reduction in the incidence of falls [45,46]. Potential obstacles to an effective risk management culture are the difficulty of ensuring adherence to protocols by patients and healthcare personnel, as well as the lack of professional training in fall prevention strategies [47]. However, there is scientific evidence demonstrating that the implementation of specific preventive programs has been effective in reducing the rate of falls [48]. 

Regulatory agencies, including the Joint Commission, the Centers for Medicare and Medicaid Services, and the Agency for Healthcare Research and Quality, have focused on fall prevention strategies since the rate of falls in hospital environments could represent an indirect sign of the quality of healthcare [49,50]. Furthermore, the Joint Commission considers falls to be a sentinel event, that is, a high-severity event that occurs with patients in the hospital environment and that could lead to death or, at least, to serious physical or psychological temporary or permanent injury [51].

In Italy, a patient’s serious injury resulting from a fall is considered a sentinel event; thus, the rate of hospital falls is an indicator of the quality of the national health system. Therefore, in order to increase patient safety in the hospital environment and, consequently, to increase the quality of care provided, in December 2011 the Ministry of Health released Recommendation No. 13, “Prevention and management of patient falls in healthcare facilities”, which is the main national mandate for the reduction of falls in the hospital environment; for this reason, all healthcare facilities are required to adopt and apply it [32]. 

In our country, falls also represents a serious economic problem for health facilities, when considering the relevant costs of the rehabilitation processes related to some of the consequences of the patients’ falls. This study reviewed a series of judgments concerning compensation for non-pecuniary damage resulting from patients’ falls in hospital, in order to identify the main reasons for accepting or rejecting the plaintiff’s claim. Considering a sample of 30 episodes of falls occurring in a hospital environment, our results show that the majority of the risk factors are related to the patient’s state of health. This series of cases is in line with the scientific literature, which reports that hospital falls mainly affect older people, even more so if they are affected by neurocognitive disorders or motor deficits that compromise their walking and postural maintenance capacity [52,53,54]. 

A relevant finding that emerged from our scientific analysis concerns the distribution of acceptance or rejection of the application. When analyzing both the judgments concerning episodes of hospital falls that occurred before the issuing of Ministerial Recommendation No. 13 (before December 2011) and those occurring after that date, it is possible to note a fair distribution between accepted claims (55.55% were accepted before December 2011 and 47.62% after that date) and rejected claims (44.44% and 52.38%, respectively). In light of this data, it can be considered that, despite the fact that the protocols for preventing falls in the hospital environment have been implemented and applied, healthcare facilities continue to be equally unsuccessful in legal proceedings. These findings could suggest that preventive strategies other than the mere application of Ministerial Recommendation No. 13 could be introduced. Apart from this, it is relevant that, to the best of our knowledge, there are no studies in the literature that analyzed the rate of application of this Recommendation in the Italian hospital environment. Therefore, further analyses are needed to examine this relevant aspect, and, should a poor application of the recommendation be found, this should then lead to the introduction of strategies to improve the method of employment of the Recommendation. Alternatively, an update of this Recommendation along with the introduction of new scientific evidence could help to better identify those patients at risk of falls in the hospital environment.

An analysis of the judgments shows that almost all the reasons given for the acceptance of the case are attributable either to the defendants’ failure to provide evidence as to the existence of a fortuitous event (46.66%) or to their failure to adopt specific means of fall protection (46.66%), which in our country are especially represented by the application of the Conley scale [55], the use of protective sides to the bed, and the correct drugs management, as reported in Ministerial Recommendation No. 13. In Italy, the legal principles that regulate the hospitalization contract between the Healthcare Facility and the patient include the obligation of healthcare staff to supervise the patient’s conditions, in order to prevent him or her from causing harm to third parties or suffering harm themselves. This principle is well-established by Law No. 24/2017, which highlights the importance of safety in healthcare as both a patient’s right and a collective interest. Furthermore, it also establishes the requirement that all healthcare personnel must contribute to the activities of patient risk prevention.

In addition to this, the “hospitalization contract” is connected to the Italian legal rules governing the accountability of medical staff and medical facilities. The Italian Civil Code’s article 1218, the “liability of the debtor”, and 1228, the “liability for acts of auxiliaries”, were applied in the event of non-compliance. Based on these requirements, the healthcare facility has to show that all the specific requirements have been met or, alternatively, that there is no causal connection between the alleged breach of the guidelines and the resulting damage. On the basis of the general principles of contractual liability, the healthcare facility must prove that it has complied with the guidelines or good clinical assistance practices (patient surveillance, the adoption of specific means of protection against falls) or offer exonerating proof that the breach was caused by a factor not attributable to a lack of appropriate care. Therefore, healthcare facilities should take greater care in documenting all the preventive strategies put in place to reduce the risk of patients falling. This could help reduce the risk of losing the case in a civil lawsuit solely because of not producing data on preventive strategies that had been put in place.

However, due to the high variability with which the risk of falling can manifest itself, it is difficult for the healthcare facility to prove that it always adequately applied the most correct conduct possible for the specific case. 

This consideration would also explain the discrepancy that can be seen when comparing the judgments that we analyzed with civil proceedings carried out in Italy on the subject of professional medical liability. According to Consulcesi (an Italian company providing assistance to health professionals), in Italy, about 2/3 of the civil proceedings regarding medical malpractice will end with the rejection of the plaintiff’s claim [56]. These data are different from those of other countries, where different rates of malpractice claims with verdicts passed against clinicians are reported. In the UK, for example, some authors reported that no more than 1 in 7 adverse events results in a malpractice claim [57]. Similar results are also reported in Taiwan, where only 14 percent of claims were accepted [58].

In our analysis, only 50% of the proceedings ended with the exclusion of liability on the part of the healthcare facility.

Although our research focuses exclusively on the Italian context, which is a limitation of this study, our results are nevertheless applicable to all hospital settings. This is true from two different points of view. First, it is true from a clinical risk management perspective since risk factor identification can contribute to reducing the rate of patient falls and, thus, increase the safety of care. Second, it is also true from a professional liability perspective because better management of such episodes would lead to a relevant decrease in the rate of claims or litigation, resulting in public spending reduction.

## 5. Conclusions

In Italy, the law mandates that the assessment of health liability cases should be made solely on the basis of documentary evidence; however, it is also influenced by the judge’s subjective judgment. Thus, Law No. 24, passed in 2017, clarified the legal procedures involved in the process of health responsibility.

Our study has shown that in Italy, the rate of loss in court for healthcare structures in cases regarding death or injury resulting from a patient’s fall in a hospital environment is about 50%; the reasons for the acceptance of the claim for compensation can be traced in half of the cases to the failure of healthcare personnel to adopt fall protection equipment. No significant difference emerged between the rates of acceptance and rejection of claims before and after the issuance of Ministerial Recommendation No. 13 in December 2011. If this proactive approach seems to not really be effective in reducing the rate of claims for healthcare responsibility, it would be beneficial to educate healthcare professionals about medico-legal issues since Italian law requires hospitals to demonstrate their actions, which necessitates that hospitals maintain complete documentation regarding their preventive measures. Aside from this approach, it is noteworthy that a comprehensive analysis of the application of the Recommendation in Italian hospitals has not yet been conducted. Therefore, additional analyses are required to clarify this relevant aspect. In the event that further investigations might reveal an insufficient application of the Recommendation, this should result in the introduction of new strategies to enhance its implementation.

The combination of these considerations and our data shows how necessary it is to carry out the implementation and standardization of fall prevention strategies in the hospital environment, in order to increase the quality of health services and reduce the risk of litigation that burdens Italian hospitals.

All these actions would be ideal in order to standardize justice in terms of health liability and, consequently, in terms of patient compensation for damages, as well as to prevent pointless lawsuits and foster a more tranquil work environment for medical professionals.

## Figures and Tables

**Table 1:** Intrinsic risk factors and the pathological consequences of falls.

Injuries	N	%	Intrinsic Risk Factors	N	%
upper limb fracture	1	3.33	neurological disorders	10	33.33
pelvic fracture	3	10	advanced age	8	26.67
femur fracture	11	36.66	cognitive impairment	7	23.33
polytrauma	6	20	movement disorders	6	20
head injury	6	20	none	6	20
knee injury	1	3.33			
pinal injury	2	6.66			

**Table 2:** Acceptance and rejection of claims for compensation pre- and post-Ministerial Recommendation No. 13.

Before 2011				After 2011			
Accepted		Rejected		Accepted		Rejected	
N	%	N	%	N	%	N	%
5	55.55	4	44.44	10	47.62	11	52.38

## Data Availability

Not applicable.

## References

[1] Oliver D., Connelly J.B., Victor C.R., Shaw F.E., Whitehead A., Genc Y., Vanoli A., Martin F.C., Gosney M.A. (2007). Strategies to prevent falls and fractures in hospitals and care homes and effect of cognitive impairment: Systematic review and meta-analyses. BMJ.

[2] Thapa P.B., Brockman K.G., Gideon P., Fought R.L., Ray W.A. (1996). Injurious falls in nonambulatory nursing home residents: A comparative study of circumstances, incidence, and risk factors. J. Am. Geriatr. Soc..

[3] Rubenstein L.Z., Josephson K.R., Robbins A.S. (1994). Falls in the nursing home. Ann. Intern. Med..

[4] Schmid A.A., Wells C.K., Concato J., Dallas M.I., Lo A.C., Nadeau S.E., Williams L.S., Peixoto A.J., Gorman M., Boice J.L. (2010). Prevalence, predictors, and outcomes of poststroke falls in acute hospital setting. J. Rehabil. Res. Dev..

[5] Hendrich A., Nyhuis A., Kippenbrock T., Soja M.E. (1995). Hospital falls: Development of a predictive model for clinical practice. Appl. Nurs. Res..

[6] De Carle A.J., Kohn R. (2001). Risk factors for falling in a psychogeriatric unit. Int. J. Geriatr. Psychiatry.

[7] Cameron I.D., Murray G.R., Gillespie L.D., Robertson M.C., Hill K.D., Cumming R.G., Kerse N. (2010). Interventions for preventing falls in older people in nursing care facilities and hospitals. Cochrane Database Syst. Rev..

[8] Nurmi I., Luthje P. (2002). Incidence and costs of falls and fall injuries among elderly in institutional care. Scand. J. Prim. Health Care.

[9] Von Renteln-Kruse W., Krause T. (2007). Incidence of in-hospital falls in geriatric patients before and after the introduction of an interdisciplinary team-based fall-prevention intervention. J. Am. Geriatr. Soc..

[10] Schwendimann R., Buhler H., De Geest S., Milisen K. (2008). Characteristics of hospital inpatient falls across clinical departments. Gerontology.

[11] Neuman M.D., Silber J.H., Magaziner J.S., Passarella M.A., Mehta S., Werner R.M. (2014). Survival and functional outcomes after hip fracture among nursing home residents. JAMA Intern. Med..

[12] Church S., Robinson T.N., Angles E.M., Tran Z.V., Wallace J.I. (2011). Postoperative falls in the acute hospital setting: Characteristics, risk factors, and outcomes in males. Am. J. Surg..

[13] Van Doorn C., Gruber-Baldini A.L., Zimmerman S., Hebel J.R., Port C.L., Baumgarten M., Quinn C.C., Taler G., May C., Magaziner J. (2003). Dementia as a risk factor for falls and fall injuries among nursing home residents. J. Am. Geriatr. Soc..

[14] Krauss M.J., Evanoff B., Hitcho E., Ngugi K.E., Claiborne Dunagan W., Fischer I., Birge S., Johnson S., Costantinou E., Fraser V.J. (2005). A case-control study of patient, medication, and care-related risk factors for inpatient falls. J. Gen. Intern. Med..

[15] Woolcott J.C., Richardson K.J., Wiens M.O., Patel B., Marin J., Khan K.M., Marra C.A. (2009). Meta-analysis of the impact of 9 medication classes on falls in elderly persons. Arch. Intern. Med..

[16] Simonson W., Han L.F., Davidson H.E. (2011). Hypertension treatment and outcomes in US nursing homes: Results from the US National Nursing Home Survey. J. Am. Med. Dir. Assoc..

[17] Capezuti E., Wagner L., Brush B.L., Boltz M., Renz S., Secic M. (2008). Bed and toilet height as potential environmental risk factors. Clin. Nurs. Res..

[18] Donald I.P., Pitt K., Armstrong E., Shuttleworth H. (2000). Preventing falls on an elderly care rehabilitation ward. Clin. Rehabil..

[19] Jewell V.D., Capistran K., Flecky K., Qi Y., Fellman S. (2020). Prediction of Falls in Acute Care Using The Morse Fall Risk Scale. Occup. Ther. Health Care.

[20] Nordin E., Lindelof N., Rosendahl E., Jensen J., Lundin-Olsson L. (2008). Prognostic validity of the Timed Up-and-Go test, a modified Get-Up-and-Go test, staff’s global judgement and fall history in evaluating fall risk in residential care facilities. Age Ageing.

[21] Morse J.M., Black C., Oberle K., Donahue P. (1989). A prospective study to identify the fall-prone patient. Soc. Sci. Med..

[22] Oliver D., Britton M., Seed P., Martin F.C., Hopper A.H. (1997). Development and evaluation of evidence based risk assessment tool (STRATIFY) to predict which elderly inpatients will fall: Case-control and cohort studies. BMJ.

[23] Hendrich A.L., Bender P.S., Nyhuis A. (2003). Validation of the Hendrich II Fall Risk Model: A large concurrent case/control study of hospitalized patients. Appl. Nurs. Res..

[24] Tinetti M.E., Williams T.F., Mayewski R. (1986). Fall risk index for elderly patients based on number of chronic disabilities. Am. J. Med..

[25] Coussement J., De Paepe L., Schwendimann R., Denhaerynck K., Dejaeger E., Milisen K. (2008). Interventions for preventing falls in acute- and chronic-care hospitals: A systematic review and meta-analysis. J. Am. Geriatr. Soc..

[26] Cameron I.D., Dyer S.M., Panagoda C.E., Murray G.R., Hill K.D., Cumming R.G., Kerse N. (2018). Interventions for preventing falls in older people in care facilities and hospitals. Cochrane Database Syst. Rev..

[27] Capezzuti E., Maislin G., Strumpf N., Evans L.K. (2002). Side rail use and bed-related fall outcomes among nursing home residents. J. Am. Geriatr. Soc..

[28] Castle N.G., Engberg J. (2009). The health consequences of using physical restraints in nursing homes. Med. Care.

[29] Stevenson D.G., Studdert D.M. (2003). The rise of nursing home litigation: Findings from a national survey of attorneys. Health Aff..

[30] World Health Organization Europe What Are the Main Risk Factors for Falls amongst Older People and What Are the Most Effective Interventions to Prevent These Falls?. https://www.euro.who.int/__data/assets/pdf_file/0018/74700/E82552.pdf.

[31] Simpattie Project Final Report Safety Improvement for Patients in Europe Reporting Period FEB 2005—Feb 2007 May 2007. https://ec.europa.eu/health/ph_projects/2004/action1/docs/action1_2004_inter_19_en.pdf.

[32] Raccomandazione Per La Prevenzione E La Gestione Della Caduta Del Paziente Nelle Strutture Sanitarie. Published 1 December 2011. https://www.salute.gov.it/imgs/C_17_pubblicazioni_1639_allegato.pdf.

[33] Tinetti M.E., Inouye S.K., Gill T.M., Doucette J.T. (1995). Shared risk factors for falls, incontinence, and functional dependence. Unifying the approach to geriatric syndromes. JAMA.

[34] Medeiros de Almeida Silva F., Peralta Safons M. (2022). Mortality from falls in the elderly in the Federal District, Brazil: Characteristics and time trend, 1996–2017. Epidemiol. Serv. Saude.

[35] Salked G., Cameron I.D., Cumming R.G., Easter S., Seymour J., Kurrle S.E., Quine S. (2000). Quality of life related to fear of falling and hip fracture in older women: A time trade off study. BMJ.

[36] Stevens J.A., Lee R. (2018). The Potential to Reduce Falls and Avert Costs by Clinically Managing Fall Risk. Am. J. Prev. Med..

[37] Florence C.S., Bergen G., Atherly A., Burns E., Stevens J., Drake C. (2018). The Medical Costs of Fatal Falls and Fall Injuries among Older Adults. J. Am. Geriatr. Soc..

[38] de Vries M., Seppala L.J., Daams J.G., van de Glind E.M.M., Masud T., van der Velde N., EUGMS Task and Finish Group on Fall-Risk-Increasing Drugs (2018). Fall-Risk-Increasing Drugs: A Systematic Review and Meta-Analysis: I. Cardiovascula Drugs. J. Am. Med. Dir. Assoc..

[39] Ryba N., Rainess R. (2020). Z-drugs and Falls: A Focused Review of the Literature. Sr. Care Pharm..

[40] Carrière I., Farré A., Norton J., Wyart M., Tzourio C., Noize P., Pérès K., Fourrier-Réglat A., Ancelin M.L. (2016). Patterns of selective serotonin reuptake inhibitor use and risk of falls and fractures in community-dwelling elderly people: The Three-City cohort. Osteoporos. Int..

[41] Gambaro E., Gramaglia C., Azzollina D., Campani D., Dal Molin A., Zeppegno P. (2022). The complex associations between late life depression, fear of falling and risk of falls. A systematic review and meta-analysis. Ageing Res. Rev..

[42] Robertson M.C., Gillespie L.D. (2013). Fall prevention in community-dwelling older adults. JAMA.

[43] Dykes P.C., Burns Z., Adelman J., Benneyan J., Bogaisky M., Carter E., Ergai A., Lindros M.E., Lipsitz S.R., Scanlan M. (2020). Evaluation of a Patient-Centered Fall-Prevention Tool Kit to Reduce Falls and Injuries: A Nonrandomized Controlled Trial. JAMA Netw. Open.

[44] Dautzenberg L., Beglinger S., Tsokani S., Zevgiti S., Raijmann R.C.M.A., Rodondi N., Scholten R.J.P.M., Rutjes A.W.S., Di Nisio M., Emmelot-Vonk M. (2021). Interventions for preventing falls and fall-related fractures in community-dwelling older adults: A systematic review and network meta-analysis. J. Am. Geriatr. Soc..

[45] Close J., Ellis M., Hooper R., Glucksman E., Jackson S., Swift C. (1999). Prevention of falls in the elderly trial (PROFET): A randomised controlled trial. Lancet.

[46] Bhasin S., Gill T.M., Reuben D.B., Latham N.K., Ganz D.A., Greene E.J., Dziura J., Basaria S., Gurwitz J.H., Dykes P.C. (2020). A Randomized Trial of a Multifactorial Strategy to Prevent Serious Fall Injuries. N. Engl. J. Med..

[47] Tinetti M.E. (2008). Multifactorial fall-prevention strategies: Time to retreat or advance. J. Am. Geriatr. Soc..

[48] Tinetti M.E., Baker D.I., King M., Gottschalk M., Murphy T.E., Acampora D., Carlin B.P., Leo-Summers L., Allore H.G. (2008). Effect of dissemination of evidence in reducing injuries from falls. N. Engl. J. Med..

[49] Oh-Park M., Doan T., Dohle C., Vermiglio-Kohn V., Abdou A. (2021). Technology Utilization in Fall Prevention. Am. J. Phys. Med. Rehabil..

[50] Turner K., Staggs V., Potter C., Cramer E., Shorr R., Mion L.C. (2020). Fall prevention implementation strategies in use at 60 United States hospitals: A descriptive study. BMJ Qual. Saf..

[51] Patra K.P., De Jesus O. (2023). Sentinel Event. StatPearls [Internet].

[52] Rubenstein L.Z., Josephson K.R. (2006). Falls and their prevention in elderly people: What does the evidence show?. Med. Clin. N. Am..

[53] Muir S.W., Gopaul K., Montero Odasso M.M. (2012). The role of cognitive impairment in fall risk among older adults: A systematic review and meta-analysis. Age Ageing.

[54] Richardson J.K., Hurvitz E.A. (1995). Peripheral neuropathy: A true risk factor for falls. J. Gerontol. A Biol. Sci. Med. Sci..

[55] Pellicciari L., Piscitelli D., Caselli S., La Porta F. (2019). A Rasch analysis of the Conley Scale in patients admitted to a general hospital. Disabil. Rehabil..

[56] (2019). Consulcesi Data. The Numbers of the Doctor-Patient Legal Dispute. https://www.consulcesi.it/blog/tutele_diritti_medico/i-numeri-del-contenzioso-legale-medici-pazienti.

[57] Oyebode F. (2013). Clinical errors and medical negligence. Med. Princ. Pract..

[58] Hwang C., Wu C., Cheng F., Yen Y., Wu K. (2018). A 12-year analysis of closed medical malpractice claims of the Taiwan civil court: A retrospective study. Medicine.

